# A breast cancer PDX collection enriched in luminal (ER ^+^) tumors and young premenopausal patients to identify new therapeutic strategies for high‐risk patients^†^

**DOI:** 10.1002/path.6418

**Published:** 2025-03-27

**Authors:** Paola Defilippi, Francesca Nigrelli, Pietro Arina, Daniela Taverna, Vincenzo Salemme

**Affiliations:** ^1^ Department of Molecular Biotechnology and Health Sciences University of Torino Torino Italy; ^2^ UCL, Bloomsbury Institute of Intensive Care Medicine, Division of Medicine University College London London UK

**Keywords:** breast cancer, PDX, CDK4/6 inhibitors

## Abstract

Breast cancer includes a group of neoplasms originating from mammary gland epithelial cells caused by a variety of genetic alterations, with different responses to treatments and outcomes. In clinical practice, estrogen receptor (ER), progesterone receptor (PR), and human epidermal growth factor receptor 2 (HER2) expression guides the distinction between luminal A (ER^+^, PR^+^, HER2^−^), luminal B (ER^+^, PR^+^, HER2^+/−^), and triple‐negative (ER^−^, PR^−^, HER2^−^), each with a distinct biological behavior and clinical and therapeutic implications. Approximately 11% of breast cancers are diagnosed in young premenopausal women aged 20–49 years. Patient‐derived xenografts (PDXs) offer a powerful solution to integrate personalized medicine and novel therapeutic agents. Dr Belletti's group recently developed a PDX biobank composed of 26 PDX lines highly enriched in luminal A and B and young premenopausal breast cancer patients. The bank faithfully recapitulates the characteristics of the original tumors. A major focus of their research was to exploit these PDXs to assess the resistance to CDK4/6 inhibitors (CDK4/6i), which are critical in managing advanced luminal breast cancers. Their effort addresses a significant gap in existing PDX models, which are limited for these patient subgroups, thereby enabling deeper insights into tumor biology and therapeutic responses in these understudied populations. © 2025 The Author(s). *The Journal of Pathology* published by John Wiley & Sons Ltd on behalf of The Pathological Society of Great Britain and Ireland.

## Breast cancer classification and response to therapy

Breast cancer (BC) is the second leading cause of cancer death among women overall [1]. Luminal BC (LBC), which accounts for roughly 70% of BC cases [2], is further classified into two subtypes: LBC A: ER^+^/PR^+^, low proliferation, and generally HER2^−^; and LBC B: ER^+^/PR^+^, higher proliferation, with some HER2^+^ cases. The HER2^+^ subtype, derived from the amplification of the *HER2* oncogene, comprises 15–20% of all BCs; the remaining 15–20% represents triple‐negative (TN) BC, which lacks ER, PR, and HER2. Moreover, premenopausal patients under 40 years of age likely present with more aggressive disease subtypes, such as TNBC and HER2^+^ BC [3]. The LBC histotype remains by far the most common, with an increased aggressiveness. Young patients display a greater likelihood of hereditary BC due to mutations in genes such as *BRCA1* and *BRCA2*.

In terms of therapy, HER2‐targeted therapies (e.g. trastuzumab, pertuzumab) are effective in HER2^+^ tumors, while in TNBC the lack of actionable targets limits the efficacy of targeted therapies, with chemotherapy being the primary treatment option. The standard treatment of LBC A/B includes endocrine therapy targeting ER to inhibit tumor growth, with selective estrogen receptor modulators (tamoxifen), aromatase inhibitors (letrozole and anastrozole), and selective estrogen receptor degraders (fulvestrant and elacestrant). Moreover, combined with endocrine therapy, CDK4/6 inhibitors (CDK4/6i) such as palbociclib, ribociclib, or abemaciclib are pivotal for targeting cell cycle progression in advanced LBC [4]. Chemotherapy (anthracyclines, taxanes, and platinum) is often used for high‐risk or advanced LBC, especially LBC B. Despite these specific therapeutic treatments, chemoresistance remains a significant barrier to effective treatments of advanced or aggressive LBC. The mechanisms of resistance include mutations in genes such as *ESR1* (encoding ER), *PIK3CA*, and *TP53* contributing to therapy resistance. Resistance to endocrine therapy often correlates with cross‐resistance to CDK4/6i and chemotherapy. Moreover, the insurgence of adaptive pathways with the activation of compensatory pathways, such as PI3K/AKT/mTOR and MYC, enables tumors to bypass therapeutic blockades [4]. Furthermore, tumor heterogeneity among different patients (inter‐tumor heterogeneity) and within an individual tumor (intra‐tumor heterogeneity) [5] leads to variations in tumor biology, making it challenging to generalize treatment approaches.

Interactions with the tumor microenvironment (TME), including stromal cells and immune cells, impact therapy responses and resistance [5]. Emerging strategies to overcome resistance include combination therapies: combining CDK4/6i with agents targeting PI3K/mTOR or other pathways, or exploring combinations of endocrine therapy with novel selective estrogen receptor degraders or PROTACs (proteolysis‐targeting chimeras) show promise in overcoming resistance.

## Patient‐derived xenografts in breast cancer research

Patient‐derived xenografts (PDXs) are widely utilized in basic research, drug development, and the evaluation of anti‐cancer drugs. PDXs are based on the resection of tumor tissue from patients and orthotopic implantation in immunodeficient mice (e.g. NOD/SCID or NSG mice). Tumors are engrafted and expanded for further study or drug testing [6]. PDXs generally retain the histopathological features, the intra‐tumoral heterogeneity, and the genetic landscape of the original tumor (mutations, copy number alterations, and transcriptomic profiles) across early passages. The overall predictive accuracy of PDXs is approximately 90%, underscoring their reliability as substitutes for direct patient testing, making them invaluable for studying tumor progression, identifying drug sensitivity markers, and potential drug combination strategies. PDXs are often referred to as a ‘clinical trial phase 0’ [7] as they serve as a preliminary step before phase I/II clinical trials.

By 2024, over 1,000 unique PDX BC models had been developed worldwide, covering all BC subtypes [6]. Despite their promise, several challenges remain: the establishment of a PDX requires several months and significant financial resources, which can be critical limitations for researchers and patients alike. The success rate of PDX development is relatively low and is mainly limited to highly malignant tumors, leaving many cancer patients unable to benefit from this approach. The genetic material and cellular characteristics of tumor tissues tend to change after several generations, making PDXs unsuitable for continuous amplification. Additionally, PDXs present significant challenges for studies involving cancer immunity, although humanized mice and animals with reconstituted human immune systems aim to address these limitations. Overall, ongoing scientific and technological progress is likely to enable their broader utilization in the future. These initiatives are now supported by consortia such as the PDXNet program (https://datacatalog.ccdi.cancer.gov/resource/PDXNet) (National Cancer Institute, USA) and the EurOPDX Consortium (https://europdx.eu/pdx-collection/), which brings together PDX developers across Europe [6].

## Enhanced understanding of breast cancer through a PDX biobank

Each tumor is elusive and complex to fight due to its heterogeneity, which can be addressed through the ability of PDXs to maintain biological complexity. Recently, Segatto *et al* [8] established a PDX biobank, implementing a pipeline for the collection of fresh BC specimens with optimized time from surgery to mouse implantation. The bank closely resembles key features of the original tumors, such as ER/PR expression and mutational profiles (*PIK3CA* and *TP53*), offering an invaluable tool for investigating the molecular and cellular mechanisms that contribute to BC progression and drug resistance. Although LBC is the most frequent BC subtype, it is underrepresented in terms of PDX collections, due to its low rate of engraftment. The study by Belletti's group [8] recognizes the critical role of heterogeneity in BC progression and treatment resistance. By including diverse BC subtypes (LBC A/B, HER2^+^, and TNBC), the authors achieved an overall success rate of 17% with 26 PDX lines from 151 attempts. Subtype‐specific success rates were 15% for LBC, 12% for HER2^+^, and 35% for TNBC. Moreover, by including premenopausal patients and considering factors such as age, prior treatments, and tumor grades, the PDX library captures the spectrum of BC biology. A major aspect of this work is its focus on LBC, for which PDX models are currently scarce compared with other BC subtypes. Notably, this PDX cohort may be utilized as a robust tool to investigate BC progression at both molecular and cellular levels, and to explore how different therapeutic approaches affect tumor evolution in terms of aggressiveness and resistance.

**Figure 1:**
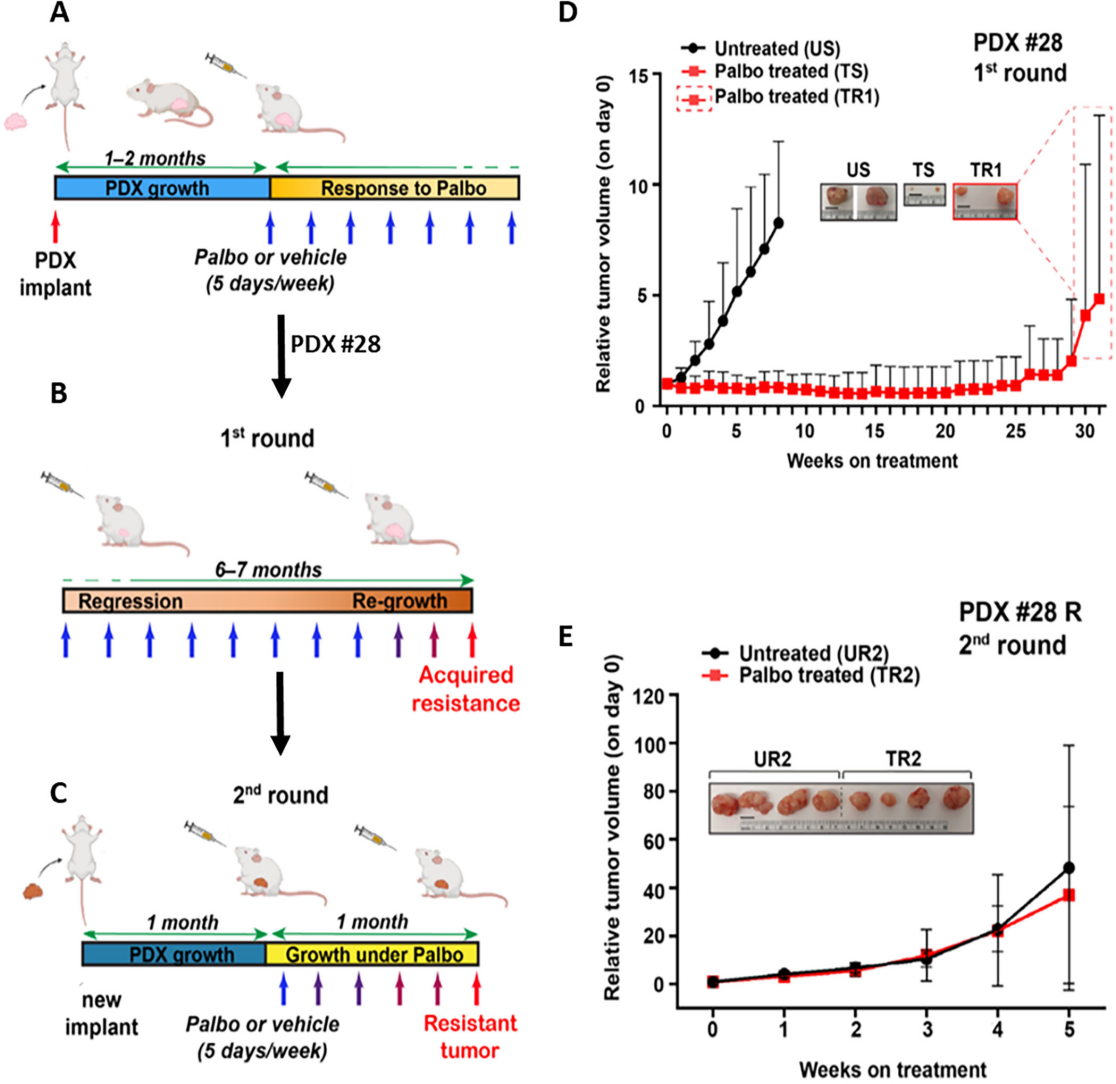
PDX repertoire as patient ‘avatars’ in assessing tumor response to combinatorial treatments. (A–C) Schematic overview of the experimental workflow: (A) PDX#28 implanted in NSG mice and left to grow for 2 months was treated with vehicle or palbociclib (palbo), which targets CDK4 and CDK6. (B) First round: PDX#28 was sensitive to palbo: under treatment, tumors started to regress, but after 6–7 months of continuous exposure to palbo, some tumors started to regrow (acquired resistance). (C) Second round: the resistant tumors were implanted in a new cohort of mice and treated again with vehicle or palbo. Tumors continued to grow in the presence of the drug, confirming the acquisition of a resistant phenotype. (D) Growth of PDX#28, treated or not with palbo (first round). The red dashed box indicates tumor regrowth following prolonged exposure to palbo. The inset displays representative tumor images. (E) Growth of palbo‐resistant tumors (TR1) emerging from the first round, untreated (UR2), or treated with palbociclib (TR2) for a second round. The inset displays representative tumor images. Modified from [8] and created in part with BioRender.com.

**Figure 2:**
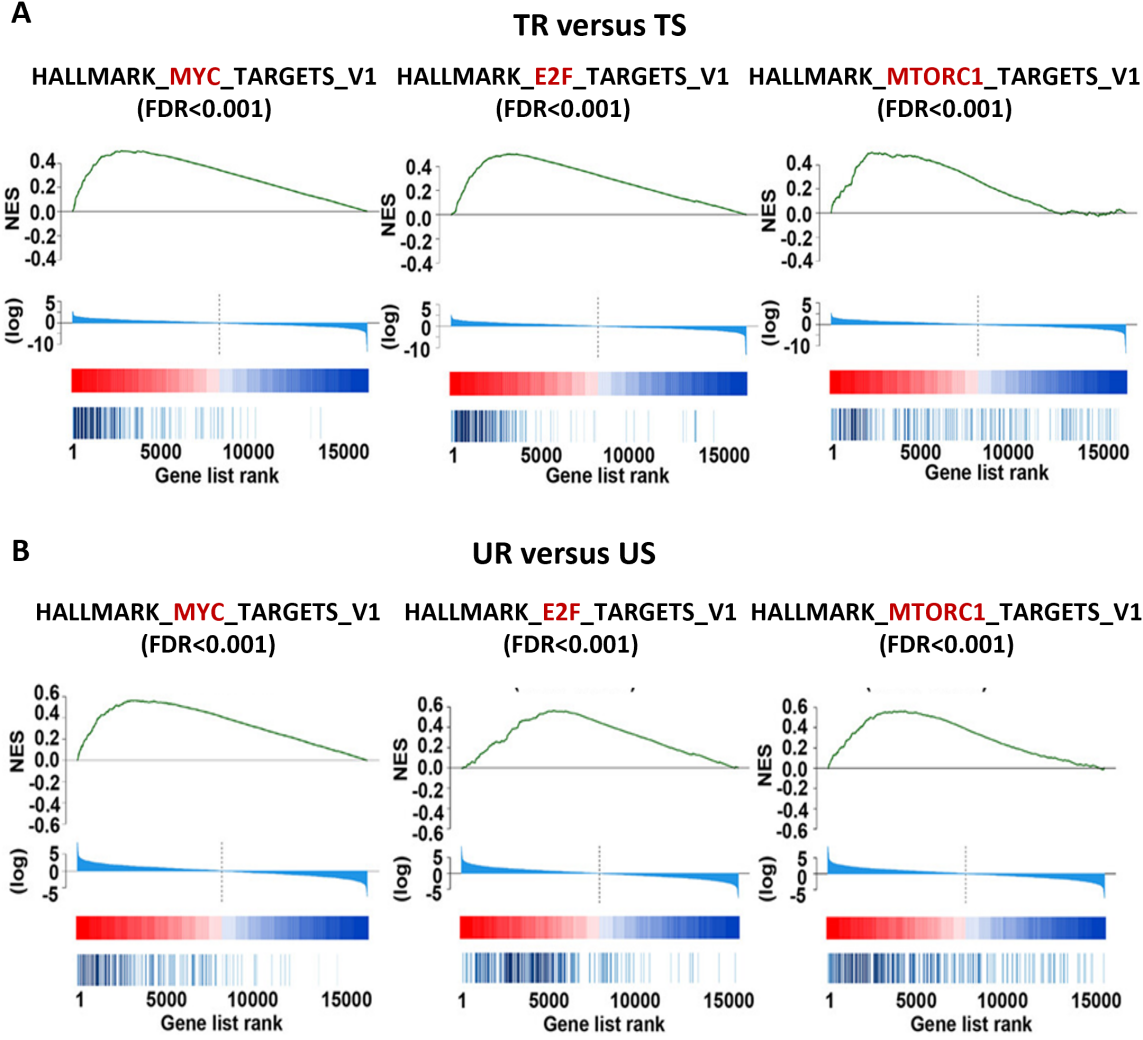
Enrichment of pathways necessary for bypassing cell cycle blockade. (A, B) Enrichment plots for the indicated pathways: (A) treated resistant (TR) versus treated sensitive (TS); (B) untreated resistant (UR) versus untreated sensitive (US). Modified from [8] and created in part with BioRender.com.

LBC is treated in the first line with endocrine therapy plus CDK4/6i [4]. However, resistance to CDK4/6i occurs frequently and represents a clinical challenge. Segatto *et al* [8] sought to elucidate the molecular mechanisms underlying CDK4/6i resistance using LBC PDXs displaying *de novo* or acquired resistance. They showed that PDX#28 under palbociclib selective pressure for over 6 months developed acquired resistance and tumors regrew (Figure 1). Pathways necessary for the bypass of cell cycle blockade imposed by palbociclib, such as MYC, E2F, and MTORC1, were greatly enriched in CDK4/6i‐resistant tumors (Figure 2). In the pre‐CDK4/6i era, to overcome the increased activation of the MTORC1 pathway during acquisition of CDK4/6i resistance, patients with advanced or metastatic LBC were treated with various FDA‐approved MTORC1 inhibitors, such as everolimus. In Segatto *et al*'s paper [8], exploiting PDX‐derived organoids (PDxO) as a pre‐clinical model in precision medicine, they found that the combination of palbociclib and the MTORC1 inhibitor rapamycin restored sensitivity in palbociclib‐resistant PDxO. Thus, the MTORC1 pathway is a promising synthetic lethal target specific for overcoming resistance to CDK4/6i in advanced LBC.

Other proliferation‐related biomarkers (e.g. MYC and E2F), stromal remodeling, ECM changes, and DNA damage repair pathways were also highlighted as potential contributors to CDK4/6i resistance, but their functional validation was not addressed. Additional analyses could be helpful to gain a more comprehensive understanding of resistance mechanisms and to identify new therapeutic strategies for patients with advanced LBC.

## Conclusion and future perspectives

Segatto *et al* [8] provide compelling evidence of the translational potential of this PDX repertoire, which fills the gap between basic biology and patients and also serves to study tumor evolution under therapeutic pressure, acting as patient ‘avatars’. By enabling the setup of ‘xeno’ clinical trials, researchers can assess tumor response to different treatments, offering molecular insights into each therapeutic arm – a path that is, of course, impossible to pursue in human patients. Looking ahead, the development of PDXs incorporating a functional human immune system could further enhance our understanding of the role of the TME and immune responses in resistance and drug sensitivity, paving the way for more effective therapeutic interventions.

Finally, artificial intelligence (AI) can enhance PDX research by improving the accuracy and efficiency of data analysis [9] through systematic comparisons and comprehensive summaries of these PDXs. AI algorithms can process vast amounts of genomic and phenotypic data from PDXs, identifying patterns and correlations that might be missed by traditional methods. This capability allows researchers to better understand tumor heterogeneity and the molecular mechanisms driving BC progression and drug resistance. AI can also facilitate the development of predictive models to forecast how different BC subtypes will respond to various treatments. AI can expedite drug discovery by thoroughly analyzing the genetic, epigenetic, transcriptional, proteomic, and/or stromal and immune‐related profiles of tumors (both patient and PDXs) and correlate these data with drug efficacy. For instance, the OncoTarget and OncoTreat platforms were developed to identify vulnerabilities across various types of cancer. By integrating data from PDX models with clinical outcomes, AI systems can help in tailoring personalized treatment strategies, potentially improving patient outcomes [9]. Furthermore, AI‐driven image analysis can enhance the evaluation of tumor growth and response to therapy in PDXs, providing precise and quantitative assessments. Lastly, the collection and standardization of data across institutions will empower a paradigm shift in healthcare and create a new era of patient‐centric care coupled with improvements in clinical outcomes [10]. Addressing these challenges is feasible with technological advancement, interdisciplinary collaboration, and commitment to enhance the translational relevance of PDX models in precision oncology.

## Author contributions statement

PD, FN, PA, DT and VS analyzed the data. PD, FN, PA, DT and VS wrote the manuscript.

## Data Availability

The data that support the findings of this study are available on request from the corresponding author. The data are not publicly available due to privacy or ethical restrictions.
